# Aerobic iron-oxidizing bacteria secrete metabolites that markedly impede abiotic iron oxidation

**DOI:** 10.1093/pnasnexus/pgad421

**Published:** 2023-12-13

**Authors:** Isabel R Baker, Sarick L Matzen, Christopher J Schuler, Brandy M Toner, Peter R Girguis

**Affiliations:** Department of Organismic and Evolutionary Biology, Harvard University, Cambridge, MA 02138, USA; Department of Soil, Water, and Climate, University of Minnesota Twin Cities, Saint Paul, MN 55108, USA; Department of Earth and Environmental Sciences, University of Minnesota Twin Cities, Saint Paul, MN 55108, USA; Department of Soil, Water, and Climate, University of Minnesota Twin Cities, Saint Paul, MN 55108, USA; Department of Earth and Environmental Sciences, University of Minnesota Twin Cities, Saint Paul, MN 55108, USA; Department of Organismic and Evolutionary Biology, Harvard University, Cambridge, MA 02138, USA

**Keywords:** iron-oxidizing bacteria, geobiology, ecophysiology, iron cycling, adaptations to oxygen

## Abstract

Iron is one of the Earth's most abundant elements and is required for essentially all forms of life. Yet, iron's reactivity with oxygen and poor solubility in its oxidized form (Fe^3+^) mean that it is often a limiting nutrient in oxic, near-neutral pH environments like Earth's ocean. In addition to being a vital nutrient, there is a diversity of aerobic organisms that oxidize ferrous iron (Fe^2+^) to harness energy for growth and biosynthesis. Accordingly, these organisms rely on access to co-existing Fe^2+^ and O_2_ to survive. It is generally presumed that such aerobic iron-oxidizing bacteria (FeOB) are relegated to low-oxygen regimes where abiotic iron oxidation rates are slower, yet some FeOB live in higher oxygen environments where they cannot rely on lower oxygen concentrations to overcome abiotic competition. We hypothesized that FeOB chemically alter their environment to limit abiotic interactions between Fe^2+^ and O_2_. To test this, we incubated the secreted metabolites (collectively known as the exometabolome) of the deep-sea iron- and hydrogen-oxidizing bacterium *Ghiorsea bivora* TAG-1 with ferrous iron and oxygen. We found that this FeOB's iron-oxidizing exometabolome markedly impedes the abiotic oxidation of ferrous iron, increasing the half-life of Fe^2+^ 100-fold from ∼3 to ∼335 days in the presence of O_2_, while the exometabolome of TAG-1 grown on hydrogen had no effect. Moreover, the few precipitates that formed in the presence of TAG-1's iron-oxidizing exometabolome were poorly crystalline, compared with the abundant iron particles that mineralized in the absence of abiotic controls. We offer an initial exploration of TAG-1's iron-oxidizing exometabolome and discuss potential key contributors to this process. Overall, our findings demonstrate that the exometabolome as a whole leads to a sustained accumulation of ferrous iron in the presence of oxygen, consequently altering the redox equilibrium. This previously unknown adaptation likely enables these microorganisms to persist in an iron-oxidizing and iron-precipitating world and could have impacts on the bioavailability of iron to FeOB and other life in iron-limiting environments.

**Significance Statement**

Iron is a required building block for all life on Earth, yet it is often a limiting nutrient due to its rapid oxidation and precipitation catalyzed by oxygen (O_2_). We found that an iron-oxidizing bacterium's exometabolite “footprint” dramatically limits net iron oxidation, allowing reduced iron (Fe^2+^) to persist for months even in the presence of O_2_. This adaptation suggests that these bacteria can have a sizeable impact on the net redox state of their environment and has likely arisen in iron-oxidizing bacteria to cope with the challenges of competing with abiotic iron oxidation. More broadly, this discovery represents further insights into the co-evolution of life and oxygen on Earth.

## Introduction

Microbes play a major role in iron's biogeochemical cycling, partly through the evolution of diverse biological strategies to scavenge, access, and transform its bioavailability. While most organisms rely on and transform iron for the purposes of assimilation (i.e. metalloprotein co-factors), there are bacteria that depend upon iron for energy metabolism. Some microorganisms, called microaerobic iron-oxidizing bacteria (FeOB), couple the oxidation of ferrous iron (Fe^2+^) to the reduction of dissolved molecular oxygen (O_2_) in aqueous systems. However, in the oxic circumneutral pH environments where these organisms live, microbially mediated iron oxidation (Eq. 1) is faced with substantial ecophysiological challenges. In particular, the abiotic oxidation of Fe^2+^ by O_2_ can be extremely rapid (1), driving Fenton-like reactions that further consume Fe^2+^ and generate harmful reactive oxygen species (ROS). In addition, the surfaces of both abiogenic and biogenic iron (oxyhydr)oxides catalyze abiotic iron oxidation (2, 3), which further reduces the electron donor pool available for metabolic consumption. Finally, abiotic and biological iron oxidation both yield insoluble iron (oxyhydr)oxides at circumneutral pH, risking cellular dysfunction or lysis if generated intracellularly or cellular entombment if produced outside of the cell.


(1) $$4Fe_{\left(\mathrm{aq}\right)}^{2+}+10H_{2}O_{\left(l\right)}+O_{2(\mathrm{aq})}\to 4\mathrm{Fe}(\mathrm{OH}{)}_{3(s)}+8H_{\left(\mathrm{aq}\right)}^{+}$$

A common argument (1, 4, 5) is that microaerobic FeOB cope with these challenges by inhabiting environments with low concentrations of dissolved O_2_, where the relative rates of abiotic iron oxidation are slower (6). However, not all FeOB live in environments where dissolved oxygen is chronically low. FeOB have been isolated from habitats such as hydrothermal vents (7), volcanic seamounts (8), coastal marine sediments (9), the pelagic water column (10–12), and the surface of the seafloor (13), where ambient O_2_ is present at or near “normoxic” concentrations (14, 15). For example, the FeOB *Ghiorsea bivora* TAG-1 (hereafter referred to as TAG-1) is a microaerobic facultative iron- and hydrogen-oxidizing zetaproteobacterium originally isolated from the Trans-Atlantic Geotraverse (TAG) hydrothermal vent site on the Mid-Atlantic Ridge, and has been detected in numerous seafloor sites throughout the Atlantic and Pacific oceans (7, 16). TAG-1 has been found living on the surfaces of polymetallic sulfides, as well as in diffuse hydrothermal flows, both of which experience dissolved oxygen concentrations up to ∼150 µM, or ∼70% of atmospheric saturation (17, 18).

Many FeOB produce visually distinct mineralized fibrals (e.g. twisted stalks, sheaths) coated with Fe(III) precipitates (19–23). TAG-1, however, forms Fe(III) aggregates that lack such distinguishing morphologies, and, relative to structures like twisted stalks, do not appear obviously biogenic (7). Given the environmental dissolved oxygen concentrations around their habitat, as well as their apparent inability to direct iron (oxyhydr)oxide deposition, TAG-1 would be expected to encounter substantial challenges in competing with abiotic iron oxidation in its environment.

It has been proposed that FeOB could excrete compounds that bind or react with iron to delay or altogether inhibit the precipitation of Fe^3+^ (20, 24–28), though this supposition has yet to be tested. Stabilizing Fe^2+^ could be achieved by compounds that either complex Fe^3+^ to increase its solubility, or that act as a redox buffer that could re-reduce Fe^3+^ to Fe^2+^ (20, 25, 26, 29, 30). Should such a mechanism be at play, it stands that any of these explanations would have a substantial impact on biogeochemical cycles that interact with iron and general redox balance in environments that host FeOB.

In this study, we sought to address the questions of how microaerobic FeOB like this compete with the abiotic oxidation of Fe^2+^ by O_2_ and how they cope with the abiotic auto-oxidation of Fe^2+^ catalyzed by solid iron (oxyhydr)oxides. Toward these ends, we investigated the biomineralizing physiology of TAG-1 via a series of laboratory incubations, geochemical analyses, and metabolomic surveys to determine whether TAG-1's iron-oxidizing physiology alters its environment to minimize the challenges of abiotic iron oxidation.

## Results

### Abiotic iron oxidation is inhibited by the exometabolites produced during biological iron oxidation

**Fig. 1.**
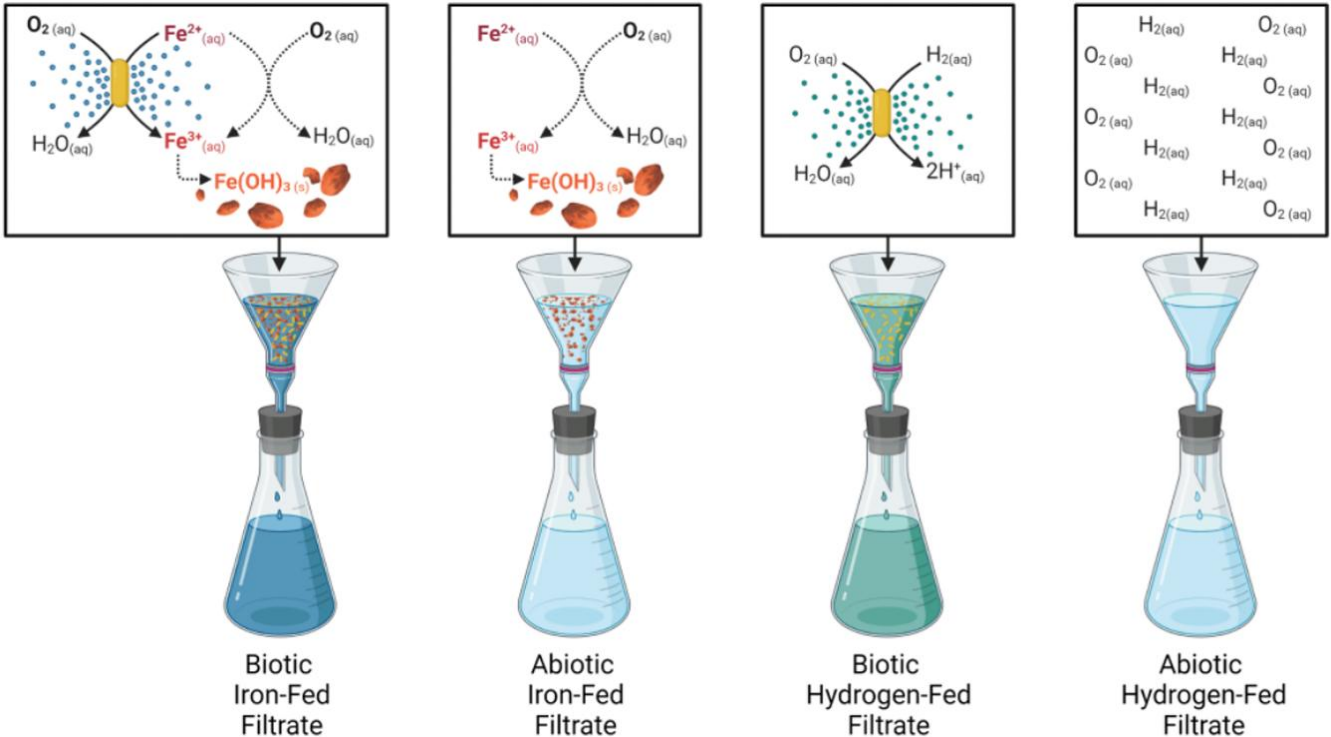
Overview of the experimental setup for filtrate incubations. Small circles represent exometabolites produced during iron oxidation or H_2_ oxidation, and ovals represent TAG-1 cells. Cultures were maintained until a specified cell density was reached, regardless of electron donor. Abiotic controls were maintained for the same amount of time it took inoculated cultures to reach the threshold cell density. Samples were then filtered, and the filtrate was either processed for downstream liquid chromatography–mass spectrometry (LCMS) or prepared for iron oxidation time-course experiments. See Materials and methods section for details.

We grew TAG-1 and uninoculated (abiotic) controls under an oxic atmosphere that approximated dissolved oxygen concentrations in the deep ocean (8% O_2_). We provided the cultures with either Fe^2+^ (iron-fed) or H_2_ (hydrogen-fed) as the sole electron donor. During this time, the cells in the biotic iron-fed and hydrogen-fed treatments would be secreting metabolites (collectively referred to as the exometabolome) into their environments as part of their normal physiological activities. To test whether the TAG-1 exometabolites influenced abiotic iron oxidation, we filtered out the cells and/or any particulates that had accumulated (>0.2 μm), leaving behind a solution of spent media and the collective exometabolome. After making these cell-free filtrates anoxic, we added fresh Fe^2+^, replenished the O_2_/CO_2_ gas headspace, and monitored iron over the course of 20 days (Fig. 1).

**Fig. 2.**
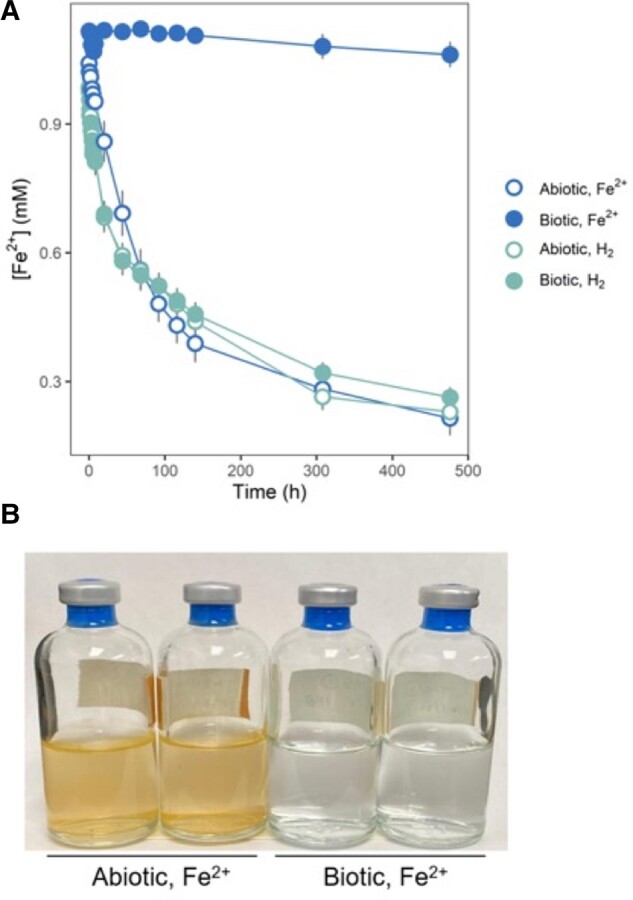
A) Fe^2+^ concentrations over time in filtrates from filtered FeCl_2_-fed and H_2_-fed cultures (TAG-1, Fe^2+^ and TAG-1, H_2_) and controls (abiotic, Fe^2+^ and abiotic, H_2_). Filtrates were made anoxic and iron levels were adjusted to 1 mM Fe^2+^ before replacing the headspace with 8% O_2_ and starting Fe^2+^ measurements. Error bars indicate standard error of the means (*n* = 4 incubations) per conditions. In some cases, error bars are smaller than the symbols. B) Images of FeCl_2_-fed abiotic (left) and biotic (right) filtrates 10 days after adding fresh Fe^2+^ and O_2_.

We observed that over the course of the cell-free incubations, rusty orange-red precipitates formed in the cell-free abiotic filtrates and biotic H_2_-fed filtrates, while the cell-free biotic FeCl_2_-fed filtrate remained visibly clear. This implies that under iron-oxidizing conditions, TAG-1's exometabolome changes environmental conditions so that iron stays in solution, either as reduced Fe^2+^ or oxidized Fe^3+^. However, quantitative measurements showed that most of the iron in biotic iron-fed filtrates remained reduced (Fe^2+^), revealing that iron oxidation was markedly inhibited in iron-fed cell-free TAG-1 filtrates (relative to all abiotic filtrates as well as the H_2_-fed TAG-1 filtrates; Fig. 2). While iron-fed abiotic filtrates, H_2_-fed abiotic filtrates, and H_2_-fed TAG-1 filtrates decreased to ∼20% of their original Fe^2+^ concentration after 20 days in the presence of O_2_, the iron-fed TAG-1 filtrates maintained ∼95% of their initial Fe^2+^ concentration after the same period of time.

Based on these measurements, TAG-1's iron-oxidizing exometabolome increases the half-life of Fe^2+^ in dysoxic seawater from 3.3 days (abiotic half-life) to 335.5 days (∼11 months) in the presence of O_2_. These observations are inconsistent with the hypothesis that FeOB secrete a compound that increases the solubility of Fe^3+^, and the precipitation of ferric minerals in the presence of TAG-1's exometabolome (next section) further negates that as a possibility. These results do, however, support a redox buffering capacity for the compounds exuded by TAG-1 when it is oxidizing iron.

### TAG-1's iron-oxidizing exometabolome changes the trajectory of abiogenic mineralization

**Fig. 3.**
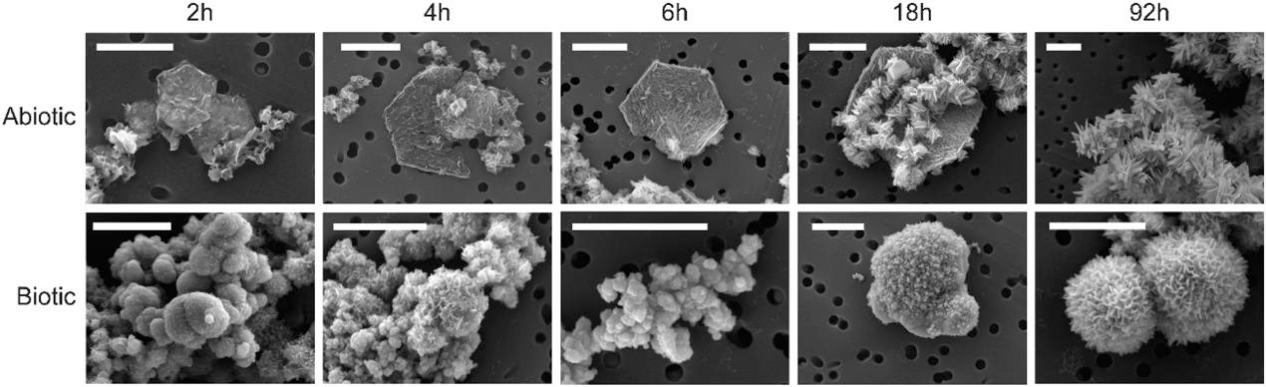
Precipitates formed in iron-fed abiotic and TAG-1 (biotic) filtrates over 92 h in the presence of O_2_. SEM images were obtained from samples that had been rinsed with anoxic water, air-dried in an anoxic chamber, and coated with Pt:Pd (80:20). Scale bars are 1 μm.

Although the biotic iron-fed filtrates appeared visually clear and iron oxidation was dramatically slowed, scanning electron microscopy (SEM) revealed that there were submicron solids present, though in far lower abundances than observed in abiotic and H_2_-fed biotic filtrates. Notably, the morphology of the suspended particulates that formed in the iron-fed biotic filtrate was strikingly distinct from those that precipitated in the abiotic filtrate (Figs. 3 and S1). The minerals that formed in the abiotic filtrate often formed hexagonal scales or other straight-edged polygonal flakes. By 92 h, most precipitates in the abiotic filtrates had transformed into larger aggregates of needle-like particles (∼1 μm in length). In comparison, the biotic filtrates contained globular aggregates composed of small (∼100–500 nm) spherical particles. In general, most of the particle surfaces in the biotic filtrate remained smooth (at 40,000× magnification) until the 92-h time point, at which point they appeared to be encrusted with flake-like features reminiscent of those formed in the earlier abiotic samples.

**Fig. 4.**
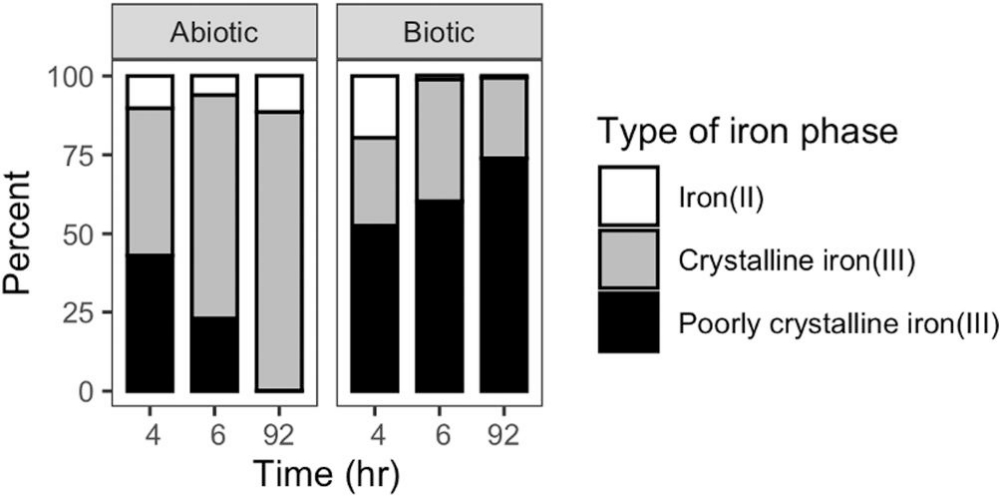
Mean percent of iron phases (grouped by mineral phase category) identified in abiotic and biotic filtrate through linear combination fitting of iron K-edge XANES spectra. See supplementary material for percentages of each phase.

Beyond gross morphological differences, X-ray fluorescence spectroscopy and spectromicroscopy revealed a higher abundance of more ordered phases (polymorphs of FeOOH: lepidocrocite, akaganeite, goethite, feroxyhyte) in the abiotic filtrate, while the biotic filtrate had a higher abundance of more poorly ordered phases (ferrihydrite- and biogenic-like iron oxyhydroxides; Fig. 4 and Table S2).

At least two well-known processes can potentially explain the influence of TAG-1's dissolved organics on mineral particle size and crystallinity. First, the dissolved organics are changing the concentration of Fe^3+^ available to form Fe(III) precipitates, and this determines which Fe(III)-bearing phase(s) will form and be stable under the experimental conditions at any given time point. Second, the presence of organics is known to decrease particle size and crystallinity and, therefore, increase the solubility of the resulting minerals. The smaller particle size and decreased crystallinity observed in the biotic filtrate are consistent with the effect of dissolved ligands on Fe(III) precipitation (31, 32) and impaired evolution (33) of precipitates from less ordered phases (e.g. ferrihydrite) to more ordered phases (e.g. lepidocrocite). Should TAG-1 be producing such ligands in order to compete with abiotic iron oxidation, the decreased structural order of the ferric iron precipitates is likely occurring as a byproduct of this process.

### Characterizing TAG-1's exometabolome

We saw that TAG-1's iron-fed exometabolome (but not its hydrogen-fed exometabolome) categorically influenced the half-life of Fe^2+^ in solution. While the primary focus of this study was to determine the fate of iron in the presence of TAG-1's exometabolome, we also chose to begin exploring the possible compounds responsible for the observed phenomenon. As such, we used an untargeted exometabolomics approach to comprehensively profile the compounds secreted by TAG-1 under iron-oxidizing conditions. The goal of applying a discovery-based technique was to capture putative target metabolites that could be responsible for the inhibition of abiotic iron oxidation in our previous experiments.

One hundred and seventy-one features were significantly (*P* < 0.01) enriched in the TAG-1 FeCl_2_-fed filtrates (relative to abiotic FeCl_2_-fed filtrates). In general, the relative abundances of these exometabolites (represented as the ratio between the spectrometric peak areas of the TAG-1 biotic media in comparison with the abiotic media) ranged from 2.023 to 175.885 (see Materials and methods for details). Three compounds were substantially more enriched in TAG-1's exometabolome, with relative abundances of 1,494.587, 1,400.446, and 838.578 (Table S1). The top 2 compounds were both identified as xanthine (with monoisotopic masses of 152.03345 and 152.03343 Da), and the next most abundant was identified as cytosine (with a monoisotopic mass of 111.04323 Da).

Surprisingly, this preliminary survey did not reveal any canonical redox buffers or iron chelators, nor were they encoded in TAG-1's genome (eq NZ_JQLW00000000.1 scanned for secondary metabolites with antiSMASH 34). This would imply that there is a component or cocktail of compounds secreted by this bacterium that have a capacity to inhibit iron oxidation that is yet to be discovered. We speculate on possible candidates in the following section.

## Discussion

Here, we showed that TAG-1's exometabolome (generated during respiratory iron oxidation) dramatically slows the net rate of abiotic iron oxidation. The half-life of Fe^2+^ was extended from 3.3 to 335.5 days at a dissolved O_2_ concentration common at the global seafloor (Fig. 2). The limited iron oxidation that did proceed resulted in the precipitation of oxidized iron minerals that were markedly less crystalline than those generated in the absence of the exometabolome, implying that these exometabolites effectively re-route ferric mineral formation. An untargeted survey of TAG-1's exometabolome revealed that xanthine and cytosine are significantly enriched relative to other exometabolites, though these compounds are not canonical iron chelators or reducers. This may indicate a novel role for these compounds in iron chemistry, and ongoing studies are looking to characterize the precise activity of xanthine and cytosine with respect to Fe^2+^.

### Toward an underlying mechanism

How is TAG-1's iron-fed exometabolome limiting abiotic iron oxidation? The major factors that contribute to net abiotic iron oxidation and its auto-catalytic nature are (i) Fe^2+^ oxidation by O_2_, (ii) the resulting ROS that oxidize three additional mols of Fe^2+^ to Fe^3+^, (iii) the concomitant precipitation of poorly soluble Fe^3+^ as solid (oxyhydr)oxides, and (iv) the surface-catalyzed Fe^2+^ oxidation caused by those precipitated minerals. Intervening in any of these processes would effectively halt the positive feedback loop of abiotic iron oxidation. For example, inhibiting Fe^3+^ hydrolysis would prevent mineral formation and surface-catalyzed Fe^2+^ oxidation, while blocking O_2_-mediated oxidation would inhibit this downstream process *as well as* the generation of Fe^2+^-oxidizing ROS that precedes it.

**Fig. 5.**
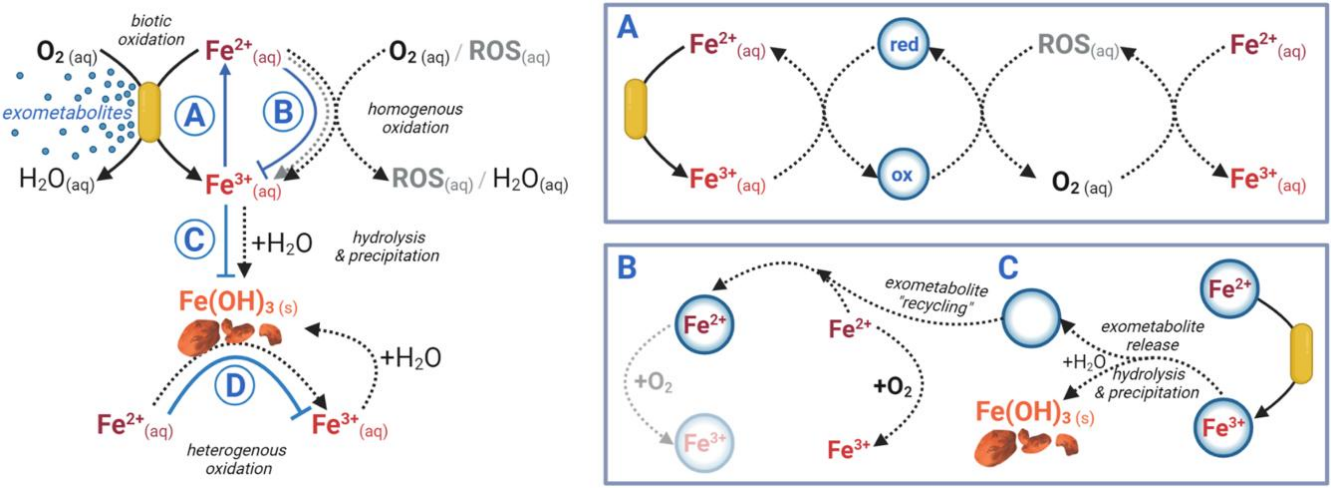
Conceptual model of possible mechanisms underlying the phenomena observed in our investigations of TAG-1's exometabolome and its impacts on iron. Dashed black and gray lines indicate abiotic processes. Solid black lines indicate biotic processes. Solid blue lines indicate hypothetical exometabolomic processes. The active agent of the exometabolome (blue circle) may be limiting net iron oxidation and iron (oxyhydr)oxide precipitation by A) re-reducing Fe^3+^ to Fe^2+^, B) inhibiting the abiotic oxidation of Fe^2+^, C) blocking Fe^3+^ hydrolysis, and/or D) by repressing surface-catalyzed heterogenous Fe^2+^ oxidation.

Xanthine and cytosine were substantially enriched in TAG-1's exometabolome, and are prime candidates for further consideration. In the absence of literature exploring these compounds' impact on iron in aqueous environments where FeOB are found, the following discussion is based on our experimental evidence, as well as previous studies, and focuses on how secreted soluble metabolites can influence dissolved iron. Organic matter (OM) has well-documented influences on iron redox cycling, environmental fluxes, solubility, and bioavailability in the environment (Table 1). Given that some OMs can chemically reduce iron and redox cycle repeatedly (29, 38, 39), TAG-1's exometabolome could be acting as a redox buffer through conventional electron transfer (Fig. 5A). Alternatively, the OM chelation of iron can shift the rates of biological and abiotic iron oxidation relative to oxidation rates as an uncomplexed ion (31, 35, 37, 40–45). Importantly, the impact that a given organic compound has on abiotic iron oxidation rates can substantially differ from the impact that same compound has on biological oxidation. For example, complexation between iron and certain organic ligands can change iron's redox potential to the point of impeding abiotic iron oxidation, though whether ligand-complexed iron is bioavailable for energy metabolism remains to be determined (46). As such, another possible mechanism could be the formation of an exometabolite-Fe^2+^ complex that is readily oxidized by TAG-1 but extremely slowly oxidized by O_2_ (Fig. 5B). Such OM-stabilized Fe^2+^ has been detected in nature, including in oxic environments like hydrothermal vent plumes and the ocean's euphotic zone (47, 48), but no causal agent had previously been identified. This would not only preserve Fe^2+^ for biologically mediated energy-yielding oxidation, but would also prevent the abiotic reaction from producing ROS that would further decrease the pool of Fe^2+^ available for biological oxidation. In this way, for every molecule of the exometabolite secreted, at least four Fe^2+^ ions would be protected from the stepwise abiotic oxidation reaction by O_2_ (Eqs. 2–5).


(2) $$Fe_{\left(\mathrm{aq}\right)}^{2+}+O_{2(\mathrm{aq})}\to Fe_{\left(\mathrm{aq}\right)}^{3+}+O_{2(\mathrm{aq})}^{∙\text{–}}$$

(3) $$Fe_{\left(\mathrm{aq}\right)}^{2+}+O_{2(\mathrm{aq})}^{∙\text{–}}+2H_{\left(\mathrm{aq}\right)}^{+}\to Fe_{\left(\mathrm{aq}\right)}^{3+}+H_{2}O_{2(\mathrm{aq})}$$

(4) $$Fe_{\left(\mathrm{aq}\right)}^{2+}+H_{2}O_{2(\mathrm{aq})}\to Fe_{\left(\mathrm{aq}\right)}^{3+}+∙OH_{\left(\mathrm{aq}\right)}+OH_{\left(\mathrm{aq}\right)}^{\text{–}}$$

(5) $$Fe_{\left(\mathrm{aq}\right)}^{2+}+∙OH_{\left(\mathrm{aq}\right)}\to Fe_{\left(\mathrm{aq}\right)}^{3+}+OH_{\left(\mathrm{aq}\right)}^{\text{–}}$$

**Table 1.** Impact of different organic matter on Fe^2+^ oxidation in the presence of O_2_.

Organic matter	[Fe^2+^]: [OM]	[O_2_] (μM)	[Fe^2+^]*_t_* _= 0_ (mM)	pH	Net impact on abiotic oxidation	Fe^2+^ half-life	Source
—	—	∼90	1	6.3	—	3.33 d	This study
EGTA	1:100	∼280	0.02	7.98	Inhibition	5.5 h	(35)
EDTA	1:100	∼280	0.02	7.98	Promotion	8.4 s	(35)
Alanine	1:100	∼280	0.02	7.98	No effect	13.8 s	(35)
Glutamic acid	1:100	∼280	0.02	7.98	No effect	14.4 s	(35)
Salicylic acid	1:100	∼280	0.02	7.14	Promotion	1.24 m^a^	(36)
Phthalic acid	1:1000	∼280	0.02	6.59	Inhibition	4.75 h^a^	(36)
Citrate	1:10	∼30	0.1	6	No effect	ND	(37)
Nitrilotriacetic acid	1:10	∼30	0.1	6	Promotion	30.8 m^a^	(37)
Pahokee peat humic acid	1:10	∼30	0.1	6	Promotion	ND	(37)
Suwannee River fulvic acid	1:10	∼30	0.1	6	No effect	ND	(37)
Exometabolome of H_2_-fed *G. bivora* TAG-1	ND	∼90	1	6.3	No effect	4.83 d	This study
Exometabolome of Fe^2+^-fed *G. bivora* TAG-1	ND	∼90	1	6.3	Inhibition	335.5 d^b^	This study

ND, not determined. ^a^Not reported but calculated from the reported rate constant(s). ^b^Calculated from the raw data.

Should biological oxidation of the Fe^2+^–exometabolite complex liberate the exometabolite, it could be recycled to complex a new Fe^2+^ ion. Alternatively, if the oxidized Fe^3+^-exometabolite complex is soluble (as is the case for many Fe^3+^–OM complexes), the Fe^3+^–exometabolite complex could be re-reduced (Fig. 5A). Depending on the dissociation rate between Fe^3+^ and the exometabolite, the hydrolysis-driven precipitation of ferric iron could be inhibited (Fig. 5C) or delayed long enough for the complex to diffuse or be advected away from the cell (Fig. 5C). OM has been reported to inhibit hydrolysis-driven ferric mineral precipitation (49). This would also suppress one of the sources of abiotic heterogeneous iron oxidation catalyzed by the surfaces of ferric minerals (Fig. 5D) (2, 50). However, given the persistence of iron in its ferrous state and the precipitation of iron that was oxidized, these two latter scenarios are less likely.

While these hypotheses involve mediating iron directly, it is also possible that other mechanisms are at play. For example, the exometabolome could consume oxygen and halt iron oxidation by eliminating its core catalyst; we find this possibility unlikely given the aerobic obligation of FeOB. Another, more plausible avenue for mediation could be shifting the chemical equilibrium such that the back reaction of the Fenton process (reverse of Eq. 2) is favored, re-reducing iron and putting a stopgap on further abiotic auto-oxidation.

Stalk-forming FeOB are thought to guide the precipitation of Fe(III)-bearing minerals onto an organic matrix secreted by the cells, and while the matrix is likely a mix of polysaccharides, the exact content is unknown (19, 21). Interestingly, although TAG-1 does not form stalks, the Fe(III) minerals we identified in the TAG-1 biotic filtrate are similar in mineral structure to those precipitated by stalk-forming FeOB (28). This suggests that certain aspects of microbial control over Fe^2+^ oxidation and mineral precipitation are not unique to stalk formation. We considered the possibility that nonstalk-forming FeOB like TAG-1 secrete an uncoordinated or monomerized version of the organic matrix used by stalk-forming FeOB. However, TAG-1's genome does not contain the known candidate gene cluster for stalk biosynthesis and export (51). Should the results from our study translate to other FeOB (like those that form stalks), we argue that this is a process separate from the adaptation underlying stalk formation.

### Candidate metabolites to explore in future studies

In the absence of any molecular smoking guns that could explain our observed phenomenon, we choose to carefully speculate on how the most abundant constituents of the exometabolome (xanthine and cytosine) could contribute to the observed hindrance of net iron oxidation. Xanthine and cytosine are nitrogen-containing heterocyclic compounds, and there is a precedent for such molecules (e.g. phenazines) to reduce Fe^3+^ to Fe^2+^ (52). It is plausible that they played a similar role in this study (e.g. cryptic iron cycling). Furthermore, both xanthine and cytosine have amino groups, which can also act as reductants for Fe^3+^. Moreover, the broader chemical class of xanthines has been shown to interact with iron during in vitro synthetic chemistry studies and inhibit corrosion in industrial settings (53). In light of these studies, as well as studies showing how nucleic acid catabolites coordinate with a number of reduced metals including iron (54–56), xanthine is a conspicuous candidate for further characterization. Likewise, although few studies have directly investigated the question of cytosine as an iron ligand under physiological conditions, cytosine has been shown to form iron–cytosine–chloride complexes under anaerobic conditions (57). There is also an extensive body of literature on the role that iron plays in stimulating damage to nucleic acids through the Fenton reaction (58, 59), and in some of these studies, it was noted that there were no visible iron oxides precipitating in the presence of cytosine, which authors presumed to the result of Fe being liganded by cytosine (59, 60).

Lastly, observations from previous studies have also shown that some bacteria secrete xanthine and cytosine under iron-*limiting* conditions, which is also consistent with our supposition that these compounds interact with iron (61–63). These previously published data, alongside the data herein, implicate xanthine and cytosine as prime candidates for the observed phenomenon, though further characterization of the mode of action, affinity, and kinetics is required to conclusively invoke these compounds in the phenomenon we observed (see above for further details). To our knowledge, there have been no direct studies on xanthine or cytosine secretion by FeOB, nor their role in limiting iron oxidation for energy metabolism. While it is possible that another, less abundant exometabolite is responsible for iron's persistence in our study, these compounds remain the prime candidates for future characterization.

## Conclusion

In this study, we sought to address the question of how microaerobic FeOB manage the abiotic oxidation of Fe^2+^ with which they compete. In focusing on the exometabolome of *G. bivora* TAG-1, a nonstalk former, we found that this iron-oxidizing bacterium's chemical footprint dramatically limits net iron oxidation, allowing Fe^2+^ to persist even in the presence of O_2_. We propose that this represents one of many adaptations that have arisen in microaerobic FeOB to cope with the challenges of a metabolism that relies on an electron donor and electron acceptor whose abiotic reaction is rapid and auto-catalytic. While further work is required to disentangle the specific mechanism at play, our findings suggest that these bacteria can have an impact on the net redox state of their environment beyond the metabolic oxidation of iron.

## Materials and methods

### Culture conditions

Cultures of TAG-1 were grown in artificial seawater for iron-oxidizing bacteria (FeOB ASW), the preparation of which is described elsewhere (8, 64). Briefly, FeOB ASW is constituted of, per liter of distilled water, 27.5 g NaCl, 5.38 g MgCl_2_, 6.78 g MgSO_4_·7H_2_O, 0.72 g KCl, 1 g NH_4_Cl, 1.4 g CaCl_2_·2H_2_O, 0.05 g K_2_HPO_4_, and 0.84 g NaHCO_3_, plus a final concentration of 0.1% MD-TMS Trace Mineral Supplement (American Type Culture Collection). After autoclaving, the pH of the media was adjusted to pH 6.3 by bubbling with filtered CO_2_:N_2_ (20:80), then amended with MD-VS Vitamin Supplement (American Type Culture Collection) to a final concentration of 0.1%.

Forty-milliliter aliquots of media were then dispensed into 60 mL combusted borosilicate glass vials. The vials were sealed with sterile butyl rubber stoppers and flushed with a filtered gas mix of O_2_:CO_2_:N_2_ (8:10:82). The headspace was refreshed and fresh electron donor was added daily. In the case of iron-fed experiments, 0.5 mM FeCl_2_ was added every day, while 0.4 cc of filtered H_2_ was added to the headspace in the hydrogen-fed treatment. Cultures and abiotic controls were kept gently rocking in the dark at 20°C.

### Experimental setup

Eight experimental replicates were set up for each abiotic and biological treatment. Abiotic vials were prepared and treated as described above. For biological treatments, vials were also prepared as described above and were inoculated with a starting cell density of 1.5 × 10^5^ cells/mL. Cells were counted daily, using methods described elsewhere (7). Briefly, glass slides with etched 10 mm circles (Electron Microscopy Sciences) were coated with 1% agarose. Ten microliters of a week-old TAG-1 culture were added on top of the solidified agarose in the center of each 10 mm circle, and the liquid was spread to encompass the area of the whole circle and allowed to dry. Once dry, 10 μL of 25 μM SYTO 13 Green Fluorescent Nucleic Acid Stain (Thermo Fisher Scientific) were added to the same area on the slide, then immediately taken for fluorescent microscopy. Cells were counted manually, with 15 fields counted per circle and 3 circles imaged per sample, for a total of 45 fields counted per sample.

Once cultures reached the late exponential phase at cell densities between 2.5 and 3 × 10^8^ cells/mL, both biological and abiotic vials were filtered for exometabolomic experiments. The contents of each vial were passed through a 0.22-μm cellulose acetate filter. The filtrates, which contained no iron (oxyhydr)oxides (at least those >0.22 μm in the case of iron-fed vials) and no cells (confirmed with fluorescent microscopy), were collected under an anoxic atmosphere. Thirty milliliters of the filtrates were transferred to 60 mL combusted borosilicate glass vials and sealed with sterile butyl rubber stoppers for subsequent experiments. At this stage, half of the replicates for each treatment (four vials each) were sacrificed for exometabolomics. The following describes the experimental setup for the remaining half of replicates used for iron-dosing experiments. Ferrous iron levels in the filtrate at this stage were assessed with the ferrozine assay (65). Given that the starting ferrous iron concentration in the abiotic, iron-fed filtrates was the highest amongst all of the treatments, we used the Fe^2+^ levels from these samples (∼1 mM Fe^2+^ according to the ferrozine assay) to determine the starting Fe^2+^ concentrations for all of our iron-dosing experiments. As such, each anoxic filtrate was injected with a final concentration of 1 mM FeCl_2_. The bottle was then immediately flushed with the low O_2_ gas mix (8% O_2_/10% CO_2_, baln. N_2_) used during the prefiltrate phase of the experiment.

Ferrous iron levels were checked immediately with the ferrozine assay and subsequently checked every 30 min for the first hour, every hour for the next 7 h, then every 12 h for the next day, every 24 h for the next week, and finally once a week for the next 2 weeks. Samples were collected for SEM and spectromicroscopy at 2, 4, 6, 20, and 92 h by applying 0.5 mL of filtrate to a 25-mm 0.2-μm track-etched Whatman Nuclepore filter (Millipore Sigma). Filters were then briefly rinsed with 0.5 mL anoxic ultrapure water and cut in half with a sterile blade. One half of each filter was submerged in a tube of anoxic ultrapure water, which was then stored at −20°C in a N_2_-purged mylar bag containing an AnaeroPak (Thermo Fisher Scientific) for downstream X-ray fluorescence microprobe and scanning transmission X-ray microscopy (STXM) sample preparation and imaging. The other half of each filter was left to air-dry under an N_2_ atmosphere in preparation for downstream SEM preparation and imaging. Sealed filtrate vials were left to gently rock at 20°C in the dark in between samplings.

### Scanning electron microscopy

SEM analyses were performed with a JEOL JSM 7900F Schottky FE-SEM at the Center for Nanoscale Systems (Laboratory for Integrated Science and Engineering, Harvard University). Dried filter pieces were adhered to aluminum stubs with double-sided carbon tape. Samples were sputter coated with 10 nm of Pt:Pd (80:20). Images were collected with an acceleration voltage of 5 kV, a probe current of ∼300 pA, and a working distance of 10–11 mm using the Lower Electron Detector. Additional SEM analyses were performed with a JEOL 6500 FE-SEM at the Characterization Facility (University of Minnesota; see Supplementary material for details).

### X-ray fluorescence spectroscopy

Iron 1 s X-ray absorption near edge structure (XANES) spectra were collected for precipitates formed in TAG-1 and abiotic filtrates at the hard X-ray microprobe beamline XFM (4-BM), National Synchrotron Light Source II, Brookhaven National Laboratory. Similar to prior studies of Fe oxyhydroxides from marine systems (66, 67), the monochromator energy was calibrated with a standard Fe foil with the inflection point set to 7,110.75 eV. Particles were located using X-ray fluorescence mapping followed by 11–24 point XANES (5 μm spot size) per sample. Fluorescence mode spectra were averaged, preedge subtracted, and postedge normalized in *Athena* (68). The resulting spectra were analyzed using linear combination fitting in *mrfitty* (69) using a publicly available reference database (70). The percentages of components of point XANES spectra were normalized to X-ray absorption intensity at 7,300 eV to more accurately represent the contribution of spots of varying intensity and then averaged to obtain a pseudo-bulk composition of all particles analyzed for a set of conditions/time points. The error is estimated to be ±5% for linear combination fitting of bulk XANES spectra (71) and is estimated to be slightly higher here due to the pseudo-bulk composition.

### Spectromicroscopy

STXM and STXM-derived XANES spectroscopy measurements were carried out on Advanced Light Source beamline 5.3.2.2 at the iron 2p edge to confirm particulate iron speciation at spatial resolutions of 30 nm. All STXM data processing was carried out using the IDL package aXis2000 (72) and Athena (68). The details are available in Supplementary material.

### Exometabolomics

Filtrates were prepared as described for previous experiments, then applied to CHROMABOND reversed phase solid-phase extraction columns (Macherey-Nagel) under an anoxic atmosphere, after the columns had first been equilibrated with liquid chromatography–mass spectrometry (LCMS)-grade methanol followed by anoxic ultrapure water. Columns were then quickly washed with water followed by elution of samples in methanol by gravity flow. Methanol samples were stored in combusted borosilicate glass vials with an N_2_ headspace and stored at −80°C before mass spectrometry. In preparation for mass spectrometry, samples were then evaporated under a flow of N_2_ and then resuspended in 50 μL 70% acetonitrile. Samples were then run on the Orbitrap ID-X Tribrid Mass Spectrometer (Thermo Fisher Scientific) at the Harvard Center for Mass Spectrometry.

The data were processed and analyzed with Compound Discoverer (Thermo Scientific, version 3.3). Peaks were extracted from MS1 data, and various adducts of the same compound were grouped together, followed by retention time alignment and gap filling between samples. The data were then median centered for normalization (i.e. the medians of all compound areas in a sample are centered around the median of all samples).

## Supplementary Material

pgad421_Supplementary_Data

## Data Availability

All data are included in the manuscript and Supplementary material.
