# Supplementary material for: Aerobic iron-oxidizing bacteria secrete metabolites that markedly impede abiotic iron oxidation
Source: PNAS Nexus. 2023 Dec 13;2(12):pgad421. doi: 10.1093/pnasnexus/pgad421 (PMC10727123; doi:10.1093/pnasnexus/pgad421)
Supplement: pgad421_Supplementary_Data [file pgad421_supplementary_data.zip › PNASNEXUS-PNASNEXUS-2023-00865R-s01.docx]

**Supplemental Information for**

Aerobic iron-oxidizing bacteria secrete metabolites that markedly impede abiotic iron oxidation.

Isabel R. Baker ^a, #, *^, Sarick L. Matzen^b^, Christopher J. Schuler^c^, Brandy M. Toner^b, c^, Peter R. Girguis^a, *^

^a^Department of Organismic and Evolutionary Biology, Harvard University, Cambridge, MA, USA; ^b^Department of Soil, Water, and Climate, University of Minnesota Twin Cities, Saint Paul, MN, USA; and ^c^Department of Earth and Environmental Sciences, University of Minnesota Twin Cities, Saint Paul, MN, USA

^#^Current address: Department of Earth and Planetary Science, Johns Hopkins University, Baltimore, MD, USA

*Corresponding authors: Isabel R. Baker and Peter R. Girguis

**Email:** ibaker5@jh.edu and pgirguis@oeb.harvard.edu

**This PDF file includes:**

Supplementary Methods

Figures S1 to S2

Legends for Tables S1 to S2

Table S2

**Other supplementary materials for this manuscript include the following:**

Table S1

**Supplementary Methods**

*Scanning electron microscopy*

Additional SEM analyses (Figure SI-1) were performed with a JEOL 6500 FE-SEM at the Characterization Facility (University of Minnesota). Dried filter pieces were adhered to aluminum stubs with double-sided carbon tape. Samples were sputter-coated with 10nm of Pt. Images were collected with an acceleration voltage of 5kV, a probe current of 8 (roughly 30pA), and a working distance of 10mm using the secondary electron image detector.

*Spectromicroscopy*

To resuspend and concentrate particles in preparation for spectromicroscopy, filter-mounted samples were vortex-mixed and sonicated in MilliQ water. The suspension was centrifuged at 13,400 rpm for 10 minutes. Supernatant was removed with a pipette, leaving approximately 20μL suspension which was again sonicated. A 2μL droplet of concentrated dispersed suspension was deposited onto a silicon nitride (Si_3_N_4_) window (Silson Ltd.) and allowed to air dry in the absence of oxygen.

Scanning transmission X-ray microscopy (STXM) and STXM-derived X-ray absorption near edge structure (XANES) spectroscopy measurements were carried out on Advanced Light Source beamline 5.3.2.2 (Berkeley, CA, USA) in November 2021. Transmission images were collected below and at the Fe 2p absorption edge, converted into optical density images (where optical density equals ln(I_0_/I), the incident X-ray intensity (I_0_) divided by the transmitted intensity through the samples (I)), and used to derive elemental maps and XANES for Fe.

The theoretical spatial and spectral resolutions of the beamline were 30 nm and ± 0.1 eV, respectively. All measurements were performed at ambient temperature and ≤ 1 atm helium. All STXM data processing was carried out using the IDL package aXis2000^33^ and Athena.^34^ Maps and stacks were aligned and converted into optical density images. Principal component and cluster analysis was used to determine representative XANES spectra which were compared to reference spectra.


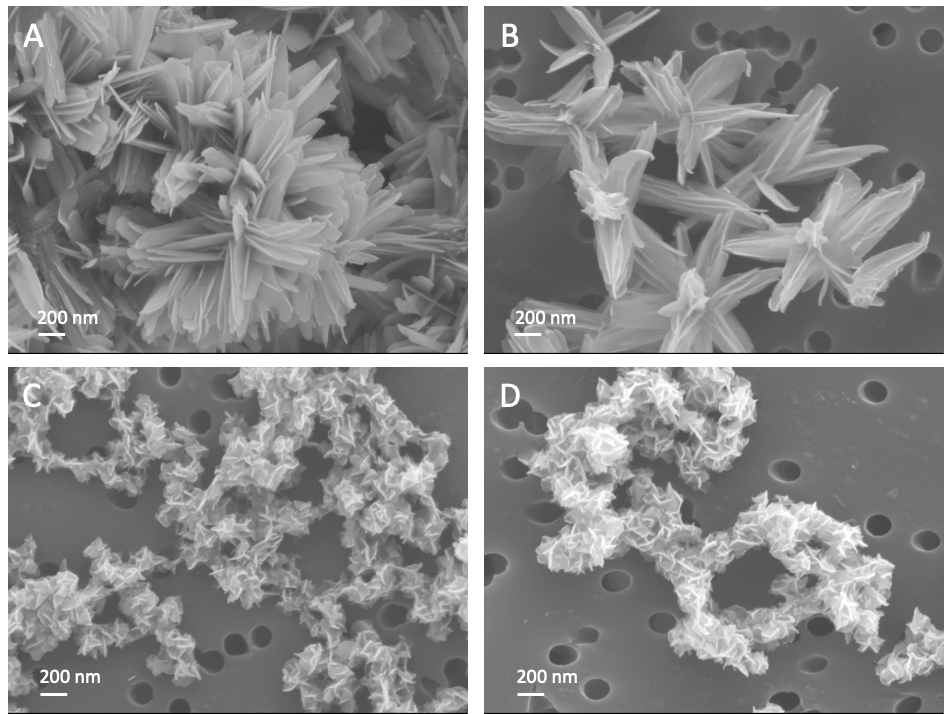


**Figure S1.** Figure SI-1. Precipitates present in iron-fed abiotic (A and B) and biotic (TAG-1) (C and D) filtrates at 92 hours in the presence of O_2_. SEM images were obtained from samples that had been rinsed with anoxic water, air-dried in an anoxic chamber, and coated with 5 nm Pt. Scale bars are 200 nm.


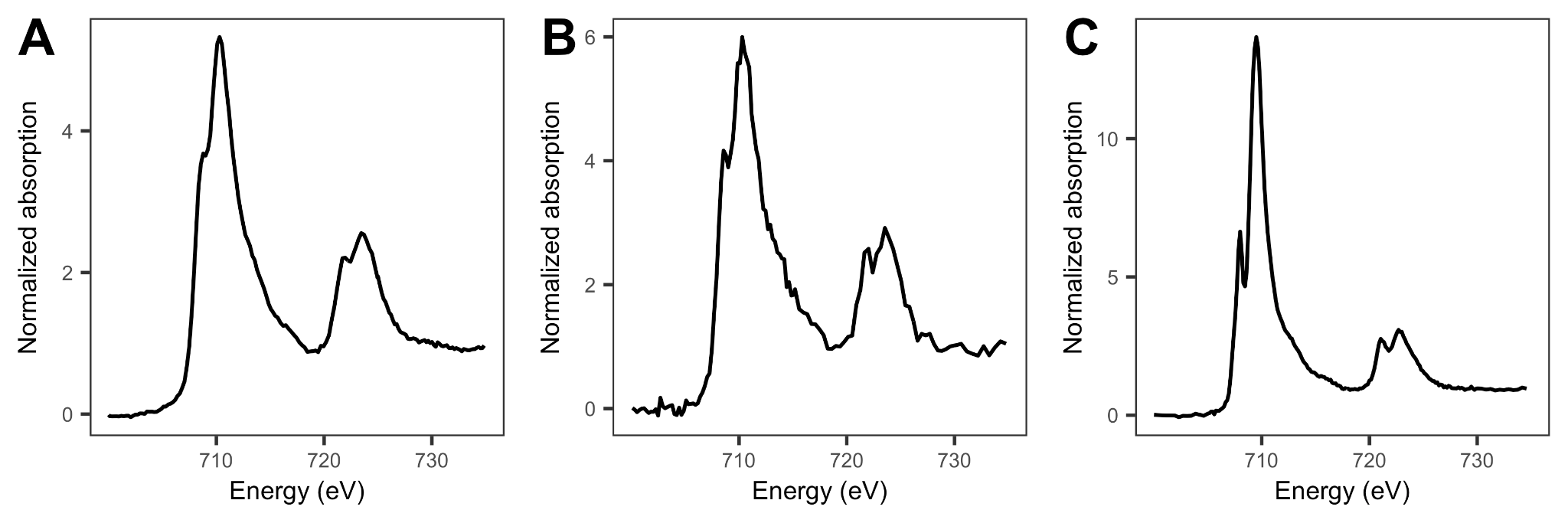


**Figure S2.** Fe 2p spectra collected from particulate matter in A) abiotic or B) biotic filtrate, compared to a C) ferrihydrite standard using scanning transmission X-ray microscopy. Spectra show that iron(III) is the dominant iron valence state at spatial resolutions of 30 nm.

**Table S1 (separate file).** Mass spectrometry data for features that were significantly (p<0.01) enriched (TAG-1:Abiotic ratio>1) in the biotic samples. Names were manually curated based on a compound’s mass, isotopes, and MSMS data. The level indicates the confidence in a compound’s name: Level 1 names are based on MSMS and retention time match with a local database, based on standards run on the same instrument at the Harvard Center for Mass Spectrometry; Level 2 indicates a name based on a MSMS match with the mzCloud mass spectral library; and Level 3 is assigned for names that also had a match in the mzCloud database, but where the MSMS data match contained some fragment discrepancies or too few fragments. Features that did not meet the criteria for any of these levels were not assigned a name. The predicted formula is based on a spectral match between the acquired MSMS data and the one present in the library; in cases where there was more than one match, the one with the highest rank (based on ppm, isotopic ratios, and MS2) is shown. The molecular weight is the mass (Da) of the monoisotopic compound. Retention time is in minutes. The MS2 column indicates whether or not there was Data-Dependent Acquisition for the preferred ion (Y for yes, N for no). The median area under the curve for the intensity of the extracted chromatogram of the main ion for that compound was used for calculating the ratio and log_2_ fold change between treatments was. The ratio and log_2_ fold change represent the general abundance of a given feature in TAG-1’s exometabolome relative to the abundance detected in the abiotic samples. P-value was computed by CompoundDiscoverer using a two-tailed student t-test.

**Table S2.** Mean plus or minus the standard error of the mean percent of iron phases (grouped by mineral phase category) identified in abiotic and biotic filtrate through linear combination fitting of iron K-edge X-ray absorption near edge structure (XANES) spectra. Standard errors are not listed when a phase was identified only once.

|  | | **Abiotic** | | | **Biotic** | | |
| --- | --- | --- | --- | --- | --- | --- | --- |
|  |  | 4hr | 6hr | 92hr | 4hr | 6hr | 92hr |
| **Crystalline phases** | Akaganeite β-FeOOH | 9.1±4.3 | 0.7 | 20±10.8 | 1.4 | 4.2±2.9 | NA |
|  | Feroxyhyte δ-FeOOH | 0.2 | NA | 1.5±0.4 | 5.2±19.1 | 6.5±12.9 | 1.6 |
|  | Goethite α-FeOOH | 3.5±3 | 13.4±2.8 | 11.5±6.4 | 4.4±9.8 | 26.1±6.3 | 8.8±4.9 |
|  | Lepidocrocite γ-FeOOH | 34±3.6 | 57±1.5 | 55.6±4.8 | 16.9±7.5 | 1.7±11.4 | 15.2±6.2 |
|  | Sum of crystalline phases | 46.8 | 71.1 | 88.5 | 27.9 | 38.6 | 25.6 |
| **Poorly crystalline phases** | Biogenic iron oxide | 39±10.6 | 17.8±3.3 | 0.1±7.1 | 40.9±7.9 | 34.8±6.4 | 60.4±4.3 |
|  | Ferrihydrite | 4±11.2 | 5.1 | NA | 11.6±7.6 | 25.5±5.9 | 13.5±5.1 |
|  | Sum of poorly crystalline phases | 43 | 22.9 | 0.1 | 52.5 | 60.3 | 73.9 |
| **Iron(II) phases** | Iron(II) oxide | 1.8 | NA | NA | NA | NA | 0.3 |
|  | Iron(II) sulfate | 7.3±3 | 3.8±1.3 | 11.5±2.7 | 8.2±7.5 | 1.3±1.8 | NA |
|  | Iron(II) in glassy silicate-1* | 0.8 | NA | NA | 7.4 | NA | NA |
|  | Iron(II) in glassy silicate-2** | 0.4 | 2.4 | NA | 4±34.8 | NA | 0.3 |
|  | Sum of iron(II) phases | 10.2 | 6.1 | 11.5 | 19.6 | 1.3 | 0.6 |

*=Iron(II) in glassy silicate (reference name Epr_basalt_glass); spectra resemble aqueous iron(II).

** =Iron(II) in glassy silicate (reference name Nkt_1g_basalt_glass); spectra resemble aqueous iron(II).
